# Giant umbilical cord in a normal preterm infant: a case report and review of the literature

**DOI:** 10.1186/s13256-022-03747-3

**Published:** 2023-01-15

**Authors:** Fariba Hemmati, Hamide Barzegar, Roya Oboodi

**Affiliations:** Neonatal Research Center, Shiraz University of Medical Sciences, Shiraz, Iran

**Keywords:** Umbilical cord anomaly, Giant umbilical cord, Thick umbilical cord, Case report

## Abstract

**Background:**

Giant umbilical cord, defined as a cord diameter of more than 5 cm, is an extremely rare malformation. There are few case reports of giant umbilical cord often associated with patent urachus duct or cystic malformation. These cases are usually managed by surgical excision and repair of patent urachus or cyst resection.

**Case presentation:**

We report the case of a 1-day-old Iranian boy with giant umbilical cord detected postnatally. The pregnancy course was uneventful, except for preterm premature rupture of the membrane and preterm delivery. There was no relevant family history. The patient was delivered by vaginal delivery with a good Apgar score. On clinical examination, the umbilical cord was very thick (about 6 cm in diameter), and huge fluctuating Wharton’s jelly was observed. Other organs were normal. During the hospital stay, the patient did not develop any complications except borderline hyperbilirubinemia, which improved with conventional phototherapy. Since the umbilical cord had no discharge and was dried, the newborn was discharged with advice for cord drying care.

**Conclusion:**

The newborn was well, and the dried umbilical stump was detached after 32 days, leaving a granulomatous structure without discharge. The patient was followed up for 4.5 months and had no problems except delayed separation of the umbilical cord.

## Background

Among umbilical cord malformations, the giant umbilical cord (GUC) is a very rare anomaly, which can be recognized by prenatal sonography or is obvious after birth. GUC is defined as a cord diameter of more than 5 cm [1], and the patent urachus duct is the most common simultaneous reported abnormality [2, 3]. The management of GUC and the need for investigation are challenging for neonatologists and pediatricians. Wildhaber *et al.* investigated the umbilical stump by histological examination, abdominal sonography, and cysto-urography [4]. These authors believed that surgery is usually required not for the condition itself but for the cause.

On the other hand, Young *et al.* stated that “most GUCs appear to be harmless, associated with normal urinary tract; hence, they may not warrant investigations” [1]. Here, we report the case of a male preterm infant with GUC, which was detected postnatally. Although the patient had delayed cord separation, the hospital course and follow-up were uneventful.

## Case presentation

**Fig. 1:**
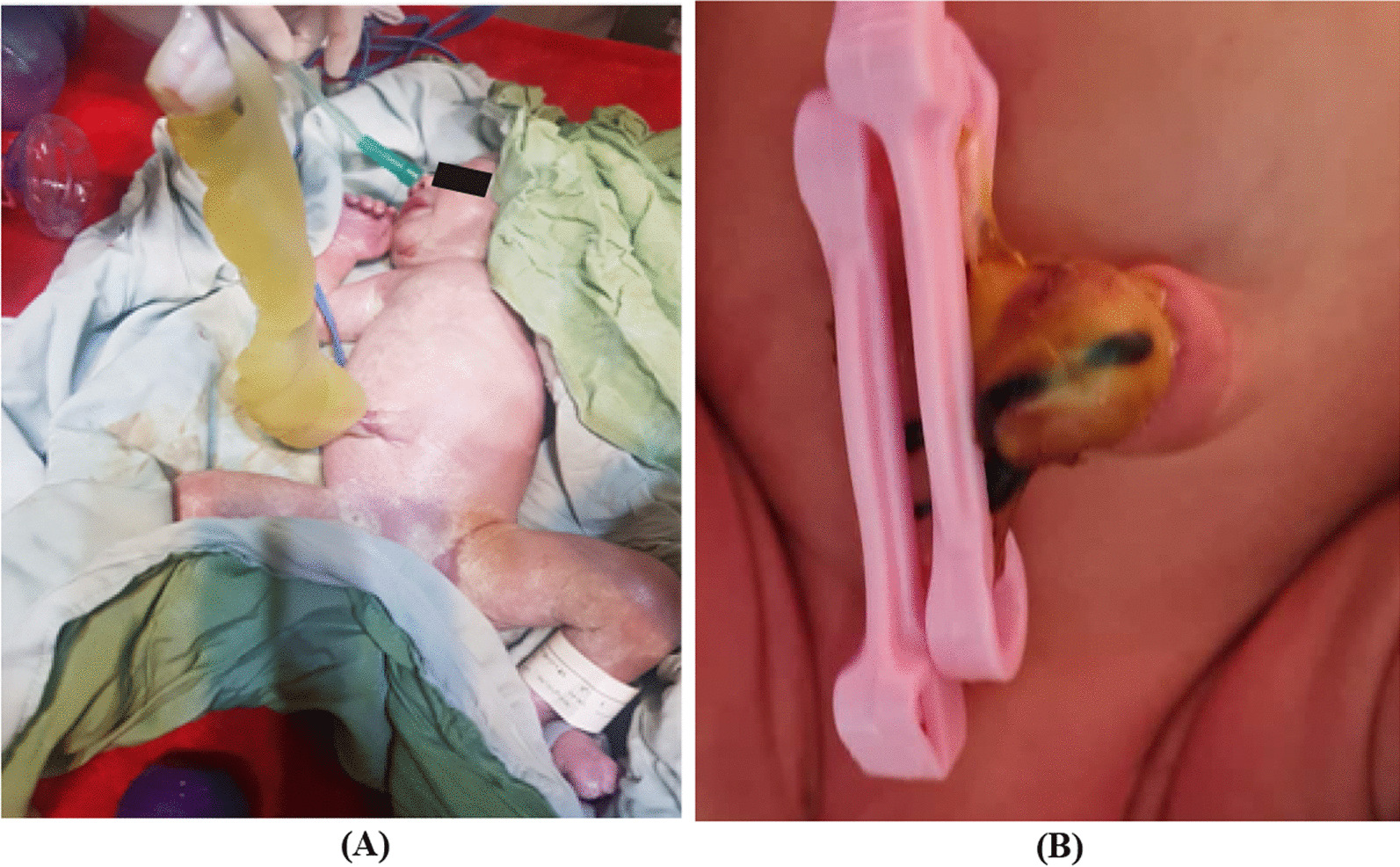
**A** Giant umbilical cord at delivery time. **B** Three days after birth

A male Iranian preterm infant was born at 32 weeks’ gestation to a 28-year-old primigravida mother. The pregnancy course was uneventful, except for preterm premature (19 hours) rupture of the membrane and preterm delivery. The patient was delivered by vaginal delivery with Apgar scores of 9 and 10 at the first and fifth minutes after birth, respectively. GUC was detected postnatally as a very thick umbilical cord (about 6 cm in diameter), its length was about 50 cm, and when cutting the umbilical cord, clear mucoid fluid and huge fluctuating Wharton’s jelly (which allowed the simple recognition of three umbilical vessels) was seen (Fig. 1A). The umbilical ring was 11 mm. The examination of other organs revealed normal results, and growth indices were appropriate for the gestational age with a birth weight of 2180 g, length of 42 cm, and head circumference of 30 cm. The infant was the first child of a nonconsanguineous couple. The mother was under medical supervision during pregnancy, and she had no complications except preterm premature rupture of the membrane and preterm delivery. Diagnosis was missed by prenatal sonography. There was no relevant family history.

**Fig. 2:**
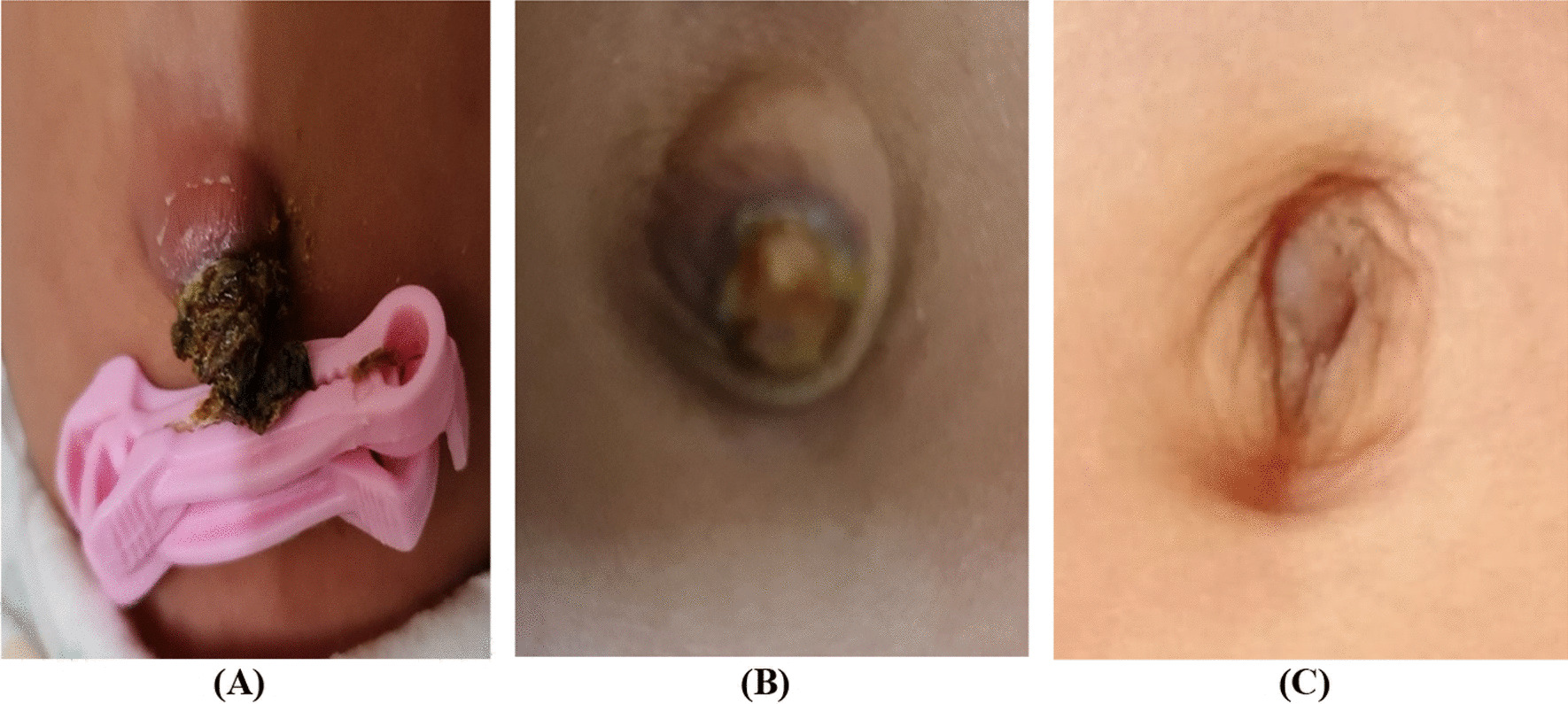
Giant umbilical cord follow-up: **A** 28 days after birth, **B** 38 days after birth, and **C** 4.5 months after birth

After birth, the newborn was transferred to the Neonatal Intensive Care Unit for prematurity care, ruling out sepsis, and managing GUC. As he was transferred from another hospital, we have no information about the placenta or pathology (as it was not requested). The patient was well during hospitalization and only developed borderline indirect hyperbilirubinemia relieved by conventional phototherapy after 2 days. The umbilical cord was relatively dried and shrunk after 3 days (Fig. 1B). Laboratory data were normal, except for mild, indirect hyperbilirubinemia (total bilirubin 11 mg/dL). Antibiotic therapy was discontinued after 48 hours because the blood culture was negative. The ultrasound findings of the kidney, ureters, and bladder and echocardiography were normal, and there was no sign of patent urachus. As the umbilical cord was relatively dried, the newborn was discharged with advice for cord drying care. On follow-up, although the newborn was well and the umbilical cord was dry, the cord was separated with delay (Fig. 2A). The dried umbilical stump was separated after 32 days, leaving a granulomatous structure without discharge (Fig. 2B). The patient was followed up for 4.5 months and had no problems except delayed separation of the umbilical cord (Fig. 2C).

## Discussion and conclusions

The umbilical cord connects the developing fetus and placenta, containing two arteries and one vein. The protector of these three vessels is Wharton’s jelly, a hydrated gel that provides flexibility and does not allow the compression of the vessels [5]. Moreover, Wharton’s jelly is a source of mesenchymal stromal cells [6]. The average normal length and diameter of the umbilical cord are 50–60 and 2 cm, respectively [7]. GUC has an abnormally huge diameter and is defined as a sonographic cross-sectional area above the 95th percentile for the gestational age [8]. The prenatal differential diagnosis includes pseudocyst, vascular malformation, umbilical hernia, omphalomesenteric duct remnant, abdominal wall defects, and urachal anomalies [9]. Although GUC can be a normal finding in some fetuses [10], there are reports of thick umbilical cords in aneuploid fetuses [11].

Fetal sonography was performed on our patient, but umbilical cord abnormality was not detected prenatally. GUC was detected postnatally, which is defined as a cord diameter of more than 5 cm [1]. There are few reports of GUC cases in the literature, often associated with the patent urachal duct and umbilical cord cyst and the need for surgery [12–14]. Schaefer *et al.* reported a case of GUC caused by retrograde micturition with open leakage into the Wharton’s jelly through a patent urachus. They recommended that diffuse GUC, elevated umbilical creatinine levels, histopathological findings of allantois remnants, and umbilical urinary discharge can support the diagnosis of a patent urachus, requiring appropriate surgical management [15]. On the other hand, we presented a GUC case without any anomalies that had a benign course at birth and on follow-up visits. The only complication was the delayed detachment of the cord stump, which was probably due to the thickness of the umbilical cord. We decided on the conservative management of GUC due to the absence of patent urachus and no sign of infection or discharge; thus, GUC seemed to be a pseudocyst. The umbilical cord shrank quickly after birth. True cord cysts are derived from the embryological remnants of the allantois, while pseudocysts arise from the liquefaction of Wharton’s jelly and lack an epithelial lining [16]. Furthermore, in the presence of a true cyst and patent urachus, some data recommend its conservative management in newborns [17]. Table 1 presents some studies on GUC from 2000. We search on PubMed, Medline, and Google Scholar with the terms GUC, giant umbilical cord, umbilical cord, and umbilical urachal cyst. Articles with non-English language were excluded. In these cases, surgical treatment refers to patent urachus repair.

While most cases of GUC are associated with other malformations, our case was an isolated finding associated with normal outcomes without surgical intervention (Table 1).

**Table 1:** Literature review

Author(s)	Year	Prenatal sonography	Postnatal finding	Treatment
Schiesser *et al.* [18]	2003	A hypoechoic mass 14 × 15 × 15 mm related to the abdominal wall without any flow within it in 14-week GA	The proximal end of the umbilicus was edematous	Surgical treatment
Nobuhara *et al.* [3]	2004	Prenatal ultrasound was not done	Giant umbilical cord: length 40 cm and diameter 5 cm Ultrasonography: bladder appeared contiguous with the base of the umbilical cord	Surgical treatment
Wildhaber *et al.* [4]	2005	Two umbilical masses	Diameter of umbilicus 3 cm Lobulated gelatinous part Hypertrophic Wharton’s jelly Sonography and cysto-urography: patent urachus	Surgical treatment
Schaefer *et al.* [15]	2010	Enlarged umbilical cord	Umbilical cord: length 50 cm and diameter 8 cm	Not mentioned
Young *et al.* [1]	2016	Thickened umbilical cord	GUC measures 10 × 12 cm and has estimated weight 500 g	Surgical treatment
Haac *et al.* [12]	2017	Intrauterine cyst	Giant umbilical cord with multiple cysts	Surgical treatment
Brooks *et al.* [19]	2017	Large cystic umbilical cord	The umbilical cord was massively enlarged and cystic	Intrauterine fetal demise
Lew *et al.* [20]	2019	Reported normal	Diameter of GUC: 4 cm Pathology confirmed umbilical pseudocyst	No surgical intervention
Aihole [2]	2019	Reported normal	Giant cystic umbilical cord 10 × 20 cm	Surgical treatment
Mugarab Samedi *et al.* [14]	2020	Not mentioned	Bulky and gelatinous umbilical cord Firm reddish sinus at the base	Surgical treatment

## Data Availability

Materials and data provided in this case study are available from the corresponding author upon reasonable request.

## Abbreviations

GUC: Giant umbilical cord
PPROM: Preterm premature rupture of the membrane
NICU: Neonatal intensive care unit
